# Commensal bacteria of the lung microbiota synergistically inhibit inflammation in a three-dimensional epithelial cell model

**DOI:** 10.3389/fimmu.2023.1176044

**Published:** 2023-04-21

**Authors:** Ellen Goeteyn, Lucia Grassi, Sara Van den Bossche, Charlotte Rigauts, Yannick Vande Weygaerde, Eva Van Braeckel, Tania Maes, Ken R. Bracke, Aurélie Crabbé

**Affiliations:** ^1^ Laboratory of Pharmaceutical Microbiology, Ghent University, Ghent, Belgium; ^2^ Cystic Fibrosis Reference Centre, Department of Respiratory Medicine, Ghent University Hospital, Ghent, Belgium; ^3^ Lung Research Lab, Department of Respiratory Medicine, Ghent University Hospital, Ghent, Belgium

**Keywords:** lung microbiome, beneficial commensal, chronic lung disease (CLD), host-microbe interaction, inflammation

## Abstract

Patients with chronic lung disease suffer from persistent inflammation and are typically colonized by pro-inflammatory pathogenic bacteria. Besides these pathogens, a wide variety of commensal species is present in the lower airways but their role in inflammation is unclear. Here, we show that the lung microbiota contains several species able to inhibit activation of the pro-inflammatory NF-κB pathway and production of interleukin 8 (IL-8), triggered by lipopolysaccharide (LPS) or H_2_O_2_, in a physiologically relevant three-dimensional (3D) lung epithelial cell model. We demonstrate that the minimal dose needed for anti-inflammatory activity differs between species (with the lowest dose needed for *Rothia mucilaginosa*), and depends on the type of pro-inflammatory stimulus and read out. Furthermore, we evaluated synergistic activity between pairs of anti-inflammatory bacteria on the inhibition of the NF-κB pathway and IL-8 secretion. Synergistic anti-inflammatory activity was observed for 4/10 tested consortia. These findings indicate that various microbiota members can influence lung inflammation either alone or as a consortium. This information can contribute to a better understanding of the lung microbiota in chronic lung disease development and process, and could open up new avenues for treatment.

## Introduction

Chronic lung inflammation is the major pathophysiological trait in airway diseases such as cystic fibrosis (CF) and chronic obstructive pulmonary disease (COPD). In people living with chronic lung disease, exposure to pro-inflammatory stimuli (such as pathogenic microorganisms, pollutants, cigarette smoke, and allergens) triggers the release of cytokines by the lung mucosa, which in turn results in an excessive immune response dominated by neutrophils (1–4). Due to the persistent inflammation and impeded mucociliary clearance, an imbalance is generated between microbial migration from the upper respiratory tract and microbial elimination through host defense mechanisms, leading to a net higher bacterial load in the lungs of patients with chronic lung disease compared to healthy individuals (5–7). The diseased lower airways are typically colonized by pathogenic bacteria such as *Pseudomonas aeruginosa*, *Staphylococcus aureus*, *Haemophilus influenzae, Moraxella catarrhalis* and *Achromobacter xylosoxidans* and their role in airway inflammation is widely recognized (8–10). Besides these common pathogens, various other lung microbiota members are present in the airways of patients with chronic lung disease (8, 11). In recent years, an association was reported between the diversity and composition of the lung microbiota and disease exacerbations (5, 12–14), inflammation (15), infection susceptibility (16), and mortality (17). Nevertheless, little is known about the role of specific microbiota members in the disease process (18). In this regard, we recently reported that a non-pathogenic commensal bacterium present in the lung microbiota, i.e. *Rothia mucilaginosa*, inhibits inflammation by interfering with NF-κB pathway activation in *in vitro* and *in vivo* models of lung inflammation (19). In addition, recent reports described that other members of the lung microbiota were able to inhibit inflammation caused by *P. aeruginosa* (20, 21).

Given the central role of the NF-κB pathway in mediating the pro-inflammatory response to a broad spectrum of inflammatory stimuli (including microorganisms, pollutants, and reactive oxygen species (ROS) (22, 23)), we aimed to understand whether the lung microbiota contains other commensal bacteria that inhibit NF-κB pathway activation, and whether species with anti-inflammatory properties may act synergistically to inhibit inflammation. To this end, we isolated a collection of commensal bacterial species from the sputum of people with cystic fibrosis (pwCF) to identify which species are able to reduce inflammation triggered by pro-inflammatory stimuli. Next, consortia of the most potent anti-inflammatory bacteria were evaluated for synergistic anti-inflammatory activity. In the present study, pro-inflammatory stimuli were selected to evaluate a host-directed anti-inflammatory effect. In that way, potential growth inhibitory effects of commensal species could be excluded, which has been described previously in co-culture studies with *P. aeruginosa* (24, 25). Knowledge on how different members of the lung microbiota simultaneously interact with the host to influence inflammation can contribute to a better understanding of the function of the lung microbiota in chronic lung disease, not only in CF but also in bronchiectasis and COPD.

## Materials and methods

### Isolation of commensal bacteria from sputum, and bacterial culture conditions

Sputum samples were collected from 5 pwCF attending the CF Reference Center of Ghent University Hospital for routine follow up, including sputum cultures. The local ethics committee of the Ghent University Hospital approved the study (registration number B670201836204). Inclusion criteria were that subjects had to be 12 years or older, clinically stable, and chronically colonized with *P. aeruginosa* (26). Before collection, saliva was removed from sputum samples by washing with sterile physiological saline. Next, Sputasol (Oxoid, Basingstoke, UK) was added to decrease viscosity and the samples were shaken for 30-60 min at 250 rpm, 37°C for liquification.

Isolation of bacterial species was done by plating samples on solid media in different oxygen conditions (27). The solid media used in this study were: Colombia agar base (Neogen, Lansing, MI, US) supplemented with 5% sheep blood (Biotrading, Mijdrecht, The Netherlands), Fastidious Anaerobe Agar (Neogen), tryptone soy agar (TSA) (Neogen) supplemented with 1.5% yeast extract (Neogen), 10 µg/mL colistin sulfate (TCI), 0.5 mg/mL L-cysteine (Sigma-Aldrich, St. Louis, MO, US), 1 ng/mL vitamin K_1_ (Sigma-Aldrich) and 10 ng/mL hemin (Sigma-Aldrich), and kanamycin vancomycin laked blood agar (KVLB), consisting of tryptone soy agar (TSA, Neogen) with 0.1 μg/ml, kanamycin (TCI, Tokyo, Japan), 7.5 μg/ml, vancomycin (Sigma-Aldrich), 10 μg/ml, vitamin K_1_ (Sigma-Aldrich), 0.05 ng/ml, hemin (Sigma-Aldrich) and 5% laked horse blood (Biotrading). Three different oxygen conditions were used for 3-5 days incubation, i.e. aerobic (37°C), microaerobic (3% O_2_, 5% CO_2_ and 92% N_2_, 37°C) using a hypoxia chamber (Bactrox; Sheldon manufacturing Inc., Cornelius, Oregon, US) and anaerobic (5% CO_2_, 5% H_2_, 90% N_2_, 37°C) using an anaerobic chamber (Bactronez; Sheldon manufacturing Inc.).

Rapid identification of bacterial isolates was done using matrix assisted laser desorption/ionization time-of-flight mass spectrometry (MALDI-TOF MS) (Bruker Microflex, Bruker Daltonics, Bremen, Germany). For isolates that could not be identified at the species level through MALDI-TOF MS, Sanger sequencing of the 16S rRNA gene was performed. Following extraction of the genomic DNA using the glass-bead method (28), amplification of the variable regions of the 16S rRNA gene was done using 27F and 1522R primers. The PCR reaction contained 0.2 µM primer mix, 25 ng template DNA, 0.2 mM deoxynucleoside triphosphates and 0.65 U Taq polymerase and the PCR protocol was carried out as follows; 95°C for 2 minutes, followed by 39 cycles of 94°C for 30 seconds, 57°C for 30 seconds and 72°C for 90 seconds, with a final temperature of 72°C for 5 min. The PCR product was verified by electrophoresis (1% agarose gel), purified using the NucleoSpin Gel and PCR Clean-up kit (Macherey-Nagel, Germany) and sequenced at GATC Biotech (Eurofins Genomics, Germany). Afterwards, sequences were analysed using the Chromas software program (Technelysium Pty Ltd, Australia). Query rRNA sequences were compared to reference sequences deposited in the NCBI 16S rRNA sequences database and in the RDP (Ribosomal Database Project) database. The BLASTn tool of the NCBI database and the Sequence Match tool (SeqMatch) of the RDP database were used for this purpose. The reference sequence with the highest similarity was determined for each query sequence, and the final identity of the isolates was established based on the consistency of the results obtained with the two databases.

All species that were evaluated for anti-inflammatory activity were cultured for 16 to 24 h at 37°C at 250 rpm in suitable broth media and oxygen levels until stationary phase was reached (**Table 1**).

**Table 1:** Overview of species used in this study and their respective culture conditions.

Genus	Species	Liquid culture	Solid culture	Oxygen level
*Actinomyces*	*- A. naeslundii* *- A. oris*	BHIB	NA	aerobic/microaerobic
*Atopobium*	*- A. parvulum*	Anaerobic BHIB + 0.2% BSA	CBA + 5% sheep blood	anaerobic
*Gemella*	*- G. haemolysans* *- G. sanguinis*	BHIB + 0.2% BSA	CBA + 5% sheep blood	microaerobic
*Micrococcus*	*- M. luteus*	BHIB	NA	aerobic
*Neisseria*	*- N. subflava*	BHIB	NA	microaerobic
*Prevotella*	*- P. histicola* *- P. melaninogenica* *- P. nigrescens* *- P. oris*	anaerobic BHIB	CBA + 5% sheep blood	anaerobic
*Rothia*	*- R. mucilaginosa*	BHIB	NA	aerobic
*Streptococcus*	*- S. salivarius* *- S. sanguinis*	BHIB	NA	microaerobic
*Veillonella*	*- V. atypica*	anaerobic BHIB + 0.1% lactate	CBA + 5% sheep blood	anaerobic

BHIB, Brain Heart Infusion broth (Neogen); anaerobic BHIB, BHIB supplemented with 0.1% resazurin (Sigma-Aldrich) and 5% L-cysteine (Sigma-Aldrich); BSA, Bovine Serum Albumin (Sigma-Aldrich); NA, Nutrient Agar (Neogen); CBA, Colombia Blood Agar (Neogen).

### Three-dimensional lung epithelial cell culture model

The alveolar epithelial cell line A549 (ATCC CCL185) was cultured as an organotypic three-dimensional (3D) cell culture model using the rotating wall vessel (RWV) bioreactor technology, which mimics *in vivo* phenotypic and functional properties of the parental tissue (29–31). This previously established 3D lung epithelial model was applied for all experiments except for the evaluation of anti-inflammatory properties of lung microbiota members based on inhibition of NF-κB pathway activation (as described below) for which a 3D model of NF-κB–luciferase-transfected A549 cells (BPS Bioscience, San Diego, CA, US) was used (19). Briefly, 3D lung epithelial cultures were generated by growing monolayers in T75 flasks containing GTSF-2 medium (HyClone, Logan, UT, US) supplemented with 1.5 g/L sodium bicarbonate (Sigma-Aldrich), 10% fetal bovine serum (FBS) (Life Technologies, Carlsbad, CA, US), 2.5 mg/L insulin transferrin sodium selenite (Lonza, Basel, Switzerland) and 1% penicillin-streptomycin (Sigma-Aldrich) at 37°C, 5% CO_2_. When confluency was reached, cells were trypsinized with 0.25% trypsin-EDTA solution (Life Technologies), counted in a Hemocytometer after viability staining using 0.4% Trypan Blue solution (Sigma-Aldrich), and 2 x 10^6^ viable cells were transferred to a RWV together with 0.25 g collagen I-coated microcarrier beads (Cytodex-3 microcarrier beads, Sigma-Aldrich) in the above-described supplemented GTSF-2 medium. After 11-14 days, cell medium was changed to GTSF-2 medium without FBS and antibiotics, and a small volume was trypsinized and counted as described above to determine aggregate density. Finally, 3D cell aggregates were transferred to a 96 well plate at a density of 2.5 x 10^5^ cells per well for host-microbe interaction experiments.

### *In vitro* host-microbe interaction studies

Bacterial cultures were centrifuged (5000 rpm, 8 minutes) and resuspended in GTSF-2 without FBS before bringing them in contact with host cells. 3D cells were exposed to lung microbiota members at various targeted multiplicity of infection (MOI) ranging from MOI 50:1 to MOI 0.003:1 with or without pro-inflammatory stimulus (100 µg/mL lipopolysaccharide (LPS) (Sigma-Aldrich) or 1 mM H_2_O_2_ (Sigma-Aldrich)). For example, 1.25 x 10^7^ bacterial CFU was added to 2.5 x 10^5^ lung epithelial cells per well to obtain an MOI of 50:1, and 7.5 x 10^2^ bacterial CFU were added for an MOI of 0.003:1. Plates were incubated for 4 h at 37°C under microaerobic conditions (3% O_2_, 5% CO_2_ and 92% N_2_) in a hypoxia chamber (Bactrox; Sheldon manufacturing Inc.). After incubation, NF-κB pathway activation, cytokine measurement, cytotoxicity and bacterial association to epithelial cells were assessed as described below.

### NF-κB luciferase assay

Following incubation of 3D NF-κB–luciferase-transfected A549 cells with bacteria and/or pro-inflammatory stimuli (LPS, H_2_O_2_), the One-Step Luciferase Assay System (BPS Bioscience) was used to determine the activation of the NF-κB-pathway, according to the manufacturer’s instructions. Luminescence was measured with an EnVision microplate reader (Perkin Elmer, Waltham, MA, US). Afterwards, values were expressed as a percentage of the signal obtained when cells were incubated with the pro-inflammatory stimulus alone (LPS or H_2_O_2_).

### Cytokine measurement

Cell supernatant was collected after incubation of 3D A549 cells with lung commensals and/or pro-inflammatory stimuli (LPS, H_2_O_2_) to quantify the concentration of secreted interleukin (IL)-8. The cytokine concentration was determined using the Human IL-8 ELISA MAX Standard assay (BioLegend, San Diego, CA, US) according to the manufacturer’s protocol. Afterwards, values were expressed as a percentage of the signal obtained when cells were incubated with the pro-inflammatory stimulus alone (LPS or H_2_O_2_).

### Cytotoxicity assay

Cell viability was assessed using the lactate dehydrogenase (LDH) detection kit (Sigma-Aldrich). To avoid potential interference of bacteria with LDH activity, the intracellular LDH assay was applied (32). Briefly, 3D A549 cells were washed twice with Hank’s Balanced Salt Solution (HBSS, Life Technologies) and lysed with 1% Triton X-100 (Sigma-Aldrich) by vigorous pipetting. LDH activity was further determined as described in the manufacturer’s protocol. Afterwards, cell viability was expressed as a percentage of the uninfected control. When viability was lower than 80% and a statistically significant difference was obtained compared to the uninfected control (p< 0.05), the commensal bacterium was considered as cytotoxic (33, 34).

### Host association assay

Bacterial association with the host cells was evaluated in the presence and absence of the pro-inflammatory stimuli (LPS, H_2_O_2_) (31). After incubation, 3D A549 cells were transferred to a new plate to avoid inclusion of possible biofilm formation in the wells. Cells were washed twice with HBSS (Life Technologies) to remove bacteria that were not associated with host cells. Next, cells were treated with 1% Triton X-100 (Sigma-Aldrich) and pipetted vigorously to lyse host cells. The obtained lysate containing bacteria associated with the host cells and intracellular bacteria were quantified by plating on solid media as described in **Table 1**.

### Synergism testing

**Figure 1:**
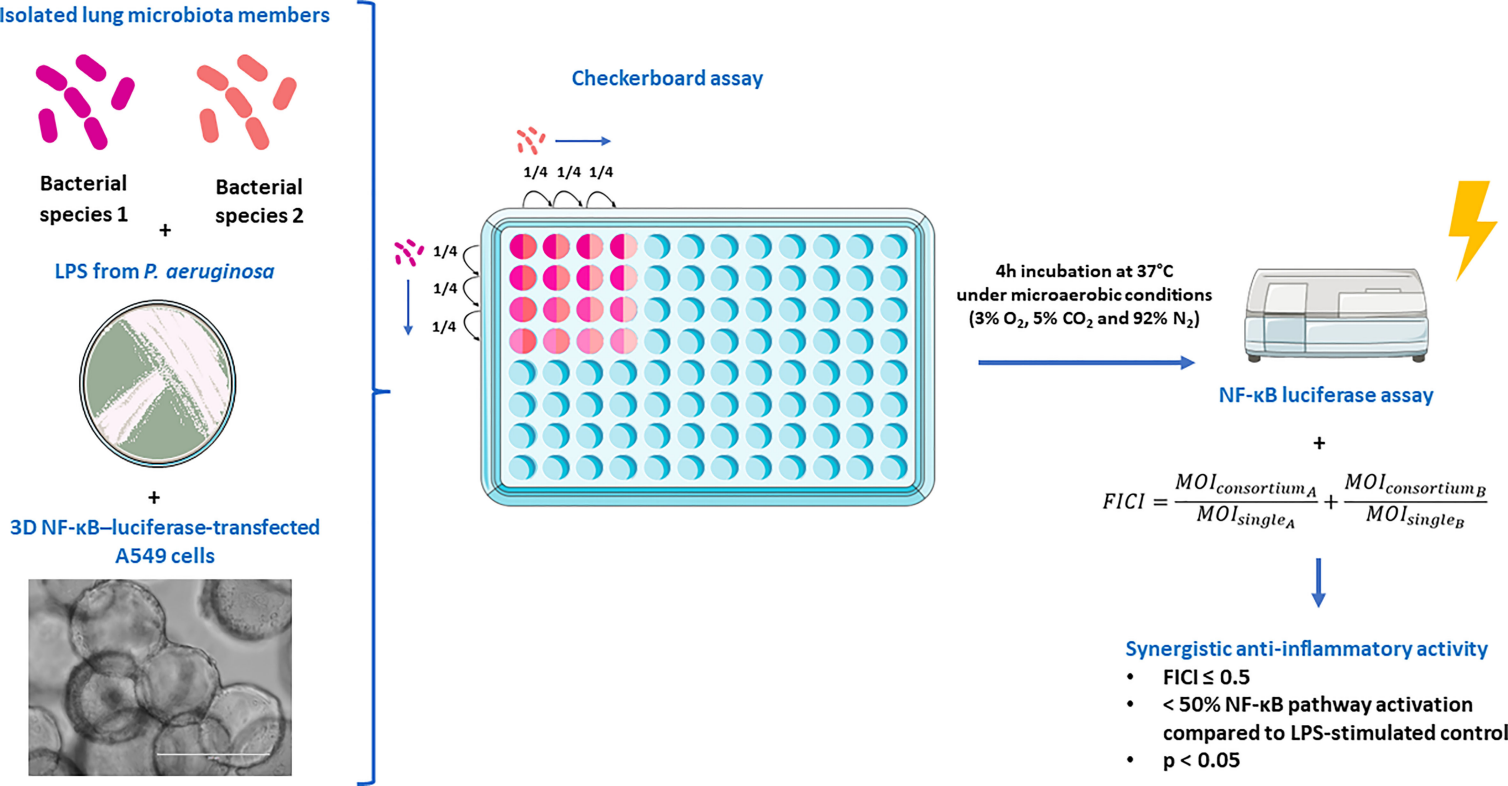
Experimental set-up to evaluate synergistic effects on the inhibition of NF-κB pathway activation in 3D NF-κB–luciferase-transfected A549 cells. The figure was partly created using the Servier Medical Art, licensed under a Creative Commons Attribution 3.0 unported license.


The experimental set-up for synergism testing is presented in **Figure 1**. Synergistic effects on the inhibition of NF-κB pathway activation in 3D NF-κB–luciferase-transfected A549 cells were evaluated by testing 10 different consortia consisting of 2 species with anti-inflammatory properties (i.e. *Actinomyces naeslundii*, *Gemella haemolysans, Prevotella nigrescens, Rothia mucilaginosa and Streptococcus sanguinis*). To this end, a modified checkerboard assay was developed, based on Stein et al., 2015 (35). Species were mixed at varying MOI (range of MOIs: 12.5 to 0.003) by applying 1:4 serial dilutions four times in a two dimensional array. Dilutions started at the mMOI_NF-κB_ of each species, which is defined as the lowest MOI with anti-inflammatory activity (i.e.< 50% NF-κB pathway activation compared to LPS-stimulated control, p< 0.05). In that way, 16 different MOI ratios were evaluated for each tested consortium. Lung epithelial cells were exposed for 4 h to 100 µg/mL LPS with or without each of the 10 consortia at varying MOI ratios, and NF-κB-pathway activation was evaluated as mentioned above. For all the MOI ratios, the fractional inhibitory concentration index (FICI) was calculated (35). This method is often used for antimicrobial drugs to evaluate if combinations of antimicrobial drugs can have a stronger effect than the sum of their effect when used alone (36). In the present study, the same principle was applied for each 2-species consortium. Hence, the following formula was used to determine the FICI:

$$FICI=\frac{{MOI}_{{consortium}_{A}}}{{MOI}_{{single}_{A}}}+\frac{{MOI}_{{consortium}_{B}}}{{MOI}_{{single}_{B}}}$$

The MOI_single_ represents the mMOI_NF-κB_ for a single species and the MOI_consortium_ is the MOI of that same species when used in the consortium. FICI values were interpreted as described by Odds 2003 (36) with modifications, i.e. FICI ≤ 0.5 indicates synergism when NF-kB pathway inactivation of > 50% is observed compared to the LPS-stimulated control (p< 0.05) (as described above); 0.5 > FICI< 4 indicates additivity or no interaction, and FICI ≥ 4 indicates antagonism. Therefore, a 2-species consortium was defined as synergistic when at least one MOI ratio fulfilled the criteria for synergism (i.e. FICI ≤ 0.5,< 50% NF-κB pathway activation compared to LPS-stimulated control, p< 0.05).

### Data analysis

All experiments were done at least in biological triplicate. Statistical analysis was performed using SPSS Statistics 27. Normal distribution was assessed with the Shapiro-Wilk test. When data was normally distributed, a one-sample t-test (NF-κB luciferase assay, cytokine measurement and LDH assay) or an independent sample t-test (bacterial host association assay) was performed. Non-normally distributed data was analyzed by a Wilcoxon signed-rank test (NF-κB luciferase assay, cytokine measurement and LDH assay) or a Mann-Whitney U test (bacterial host association assay). When multiple conditions were compared to one control, Benjamini-Hochberg correction was applied to control for multiple testing (37). Statistical significance was considered at p< 0.05.

## Results

### Isolation of lung microbiota members from sputum samples of people with CF

**Figure 2:**
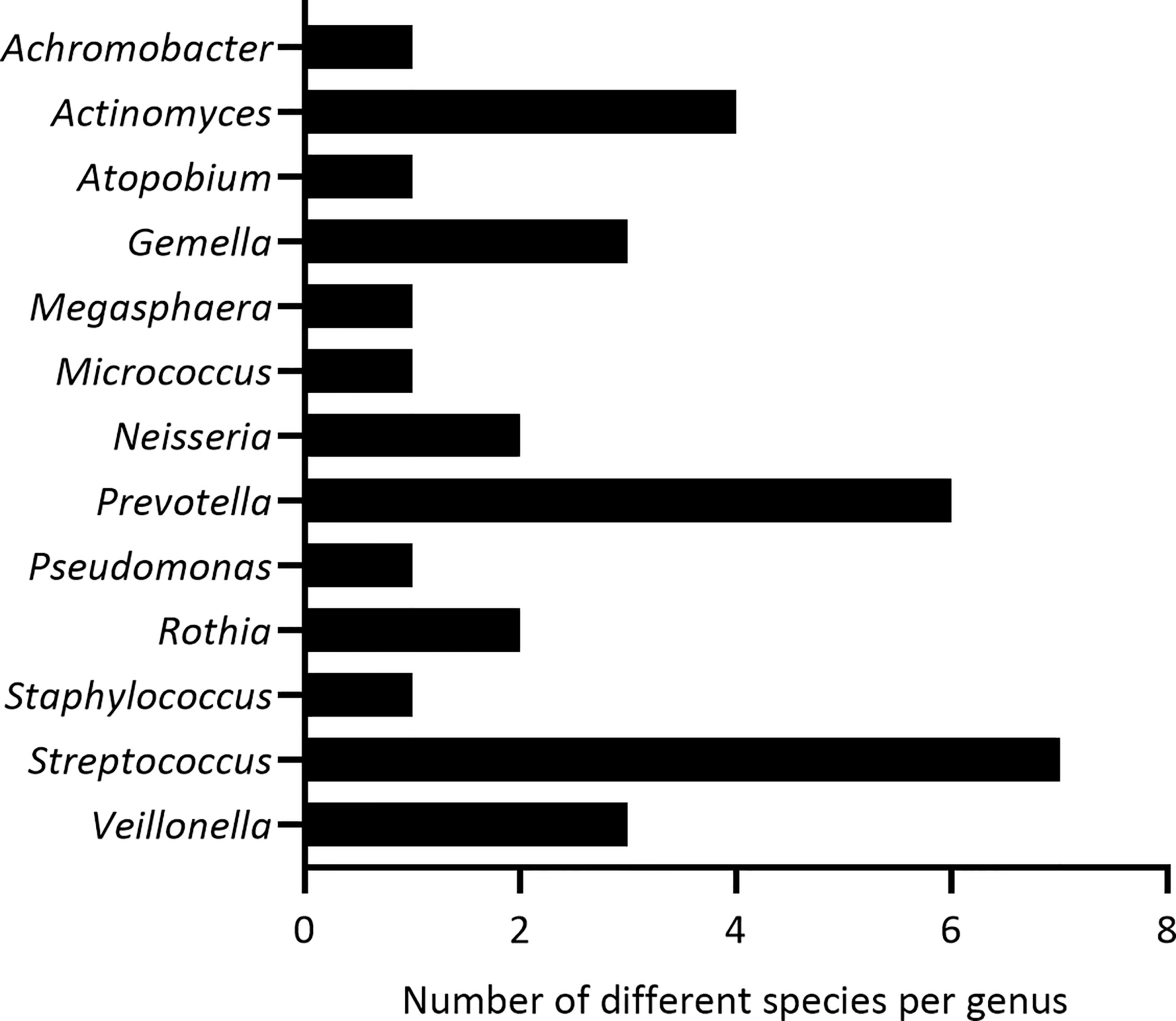
Identified genera and the number of bacterial species per genus, isolated from 5 sputum samples of pwCF.

From 5 sputum samples of adult pwCF, 33 bacterial species from 13 different genera were isolated and identified by MALDI-TOF MS or Sanger sequencing of the 16S rRNA gene (**Figure 2**). These included 3 pathogenic genera (i.e. *Achromobacter*, *Pseudomonas*, *Staphylococcus*) which were excluded for further analysis. In addition, *Megasphaera* sp. were not included for subsequent experiments since the growth media wherein all other microbiota members were cultured (**Table 1**), was not suitable for this bacterial species. This resulted in a collection of 9 non-pathogenic genera consisting of 29 species in total, from which we selected at least one species per genus to evaluate anti-inflammatory activity (15 species in total) (**Table 1**).

### The lung microbiota contains anti-inflammatory bacterial species

To evaluate whether lung microbiota members have anti-inflammatory properties, 3D cells were exposed to each commensal species in the absence or presence of LPS. This evaluation was initially done at two different MOIs (i.e. 50 and 12.5). Bacterial species were considered as anti-inflammatory when they significantly reduced LPS-triggered NF-κB-pathway activation by at least 50% at MOI 50 and/or MOI 12.5, without activating the NF-κB-pathway by itself (i.e. no significant difference between the uninfected control and cells co-cultured with commensals alone at MOI 50 or MOI 12.5). In addition, commensal species were evaluated for cytotoxicity in the 3D lung epithelial cell model.

**Figure 3:**
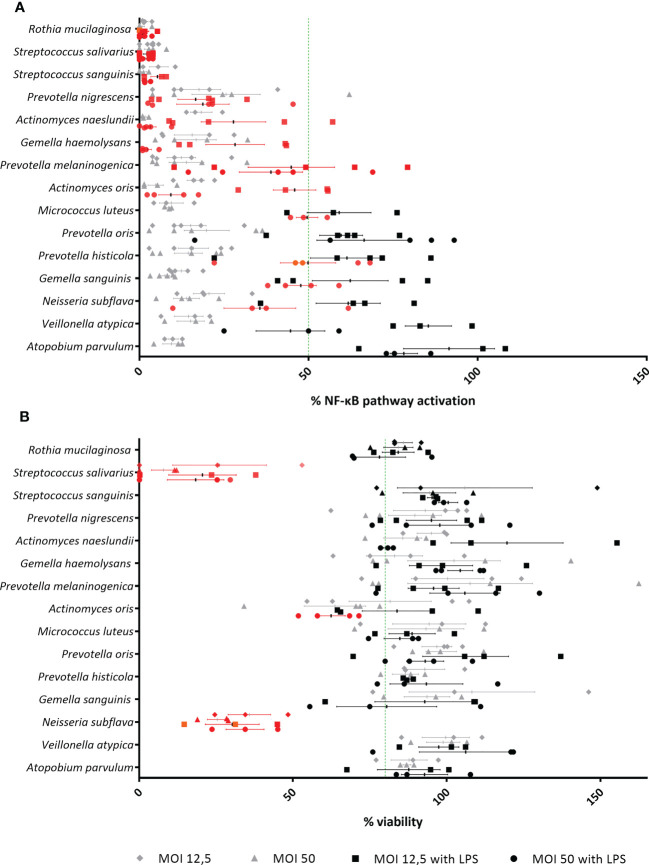
Evaluation of lung microbiota members for identification of anti-inflammatory and non-cytotoxic species in (3D) A549 (luciferase NF-κB-reporter) cells. **(A)** Isolated lung commensals at MOI 50 and MOI 12.5, with or without LPS, were screened for anti-inflammatory activity by a NF-κB luciferase assay. On the vertical axis, screened lung commensals are depicted, on the horizontal axis, the % NF-κB pathway activation compared to LPS-stimulated cells is shown. Anti-inflammatory effects were defined as;< 50% NF-κB pathway activation compared to LPS-stimulated cells as visualized by a green line, p< 0.05. Anti-inflammatory commensals are marked in red on the graph. N ≥ 3. **(B)** Isolated lung commensals at MOI 50 and MOI 12.5, with or without LPS, were screened for cytotoxicity by a LDH assay. On the vertical axis, screened lung commensals are depicted, on the horizontal axis, the % viability compared to cells cultured alone is shown. Cytotoxic effects were defined as;< 80% viability compared to cells cultured alone as visualized by a green line, p< 0.05. Cytotoxic commensals are marked in red on the graph. N ≥ 3.

Among the 15 commensal species that were evaluated, 12/15 species reduced the activation of the NF-κB pathway at a MOI 50, whereas 8/15 species reduced the NF-κB pathway activation at both MOIs (**Figure 3A**). For 3/15 species (i.e. *Actinomyces oris*, *Neisseria subflava* and *Streptococcus salivarius*), cytotoxicity was observed at a MOI of 50 (i.e. *A. oris*) or at both tested MOIs (i.e. *N. subflava*, *S. salivarius*) (**Figure 3B**). These commensals also exhibited inactivation of the NF-κB pathway, most likely as a consequence of cytotoxicity, and were excluded from this study. Hence, 9/15 species were defined as non-cytotoxic and anti-inflammatory (at MOI 12.5 and/or 50) and were evaluated further.

Finally, bacterial association with 3D cells was evaluated. Here, no significant differences were found when commensals were added to the 3D cells with or without LPS (**Supplementary Figure S1**).

### The minimal effective MOI differs between anti-inflammatory species

**Figure 4:**
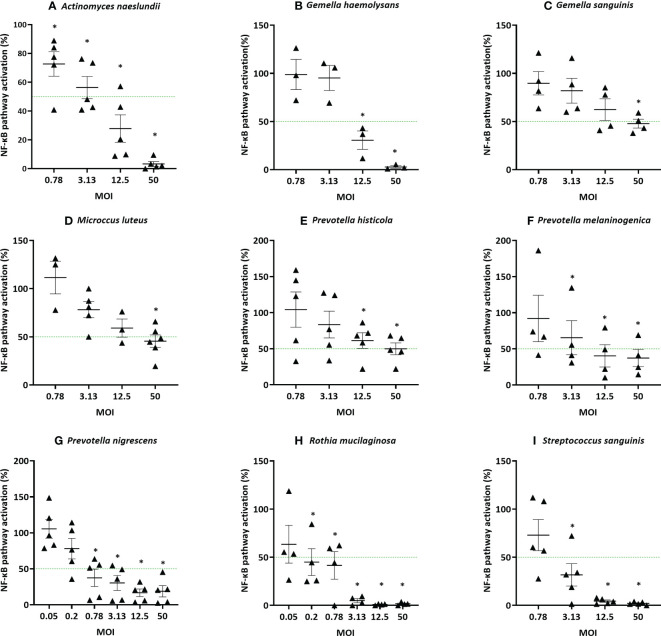
Determination of the minimal effective MOI (mMOI_NF-κB_) of commensal bacteria based on NF-κB pathway activation in (3D) A549 luciferase NF-κB-reporter cells. Selected lung commensals at different MOI were cultured with LPS and the mMOI_NF-κB_ was determined by a NF-κB luciferase assay, with **(A)** *A. naeslundii*, **(B)** *G. haemolysans*, **(C)** *G. sanguinis*, **(D)** *M. luteus*, **(E)** *P. histicola*, **(F)** *P. melaninogenica*, **(G)** *P. nigrescens*, **(H)** *R. mucilaginosa*, **(I)** *S. sanguinis*. On the vertical axis % NF-κB pathway activation compared to LPS-stimulated cells is shown, on the horizontal axis the tested MOI is depicted. The mMOI_NF-κB_ was defined as the lowest MOI with anti-inflammatory activity (i.e.< 50% NF-κB pathway activation compared to LPS-stimulated cells as visualized by a green line, p< 0,05). *: p< 0.05, n ≥ 3.

Next, anti-inflammatory and non-cytotoxic lung microbiota members were further investigated to determine their minimal effective MOI (mMOI_NF-κB_), i.e. the lowest MOI that is able to significantly decrease the LPS-triggered NF-κB-pathway activation by at least 50%. The dose that is necessary for an anti-inflammatory effect was found to differ between genera and even between species from the same genus (**Figure 4**). For example, a MOI of at least 12.5 was needed to obtain an anti-inflammatory effect for *P. melaninogenica*, whereas *P. nigrescens* was already anti-inflammatory at a MOI of 0.78. From all evaluated species and under the tested conditions, *Rothia mucilaginosa* was the most potent anti-inflammatory bacterium with a mMOI_NF-κB_ of 0.2.

**Figure 5:**
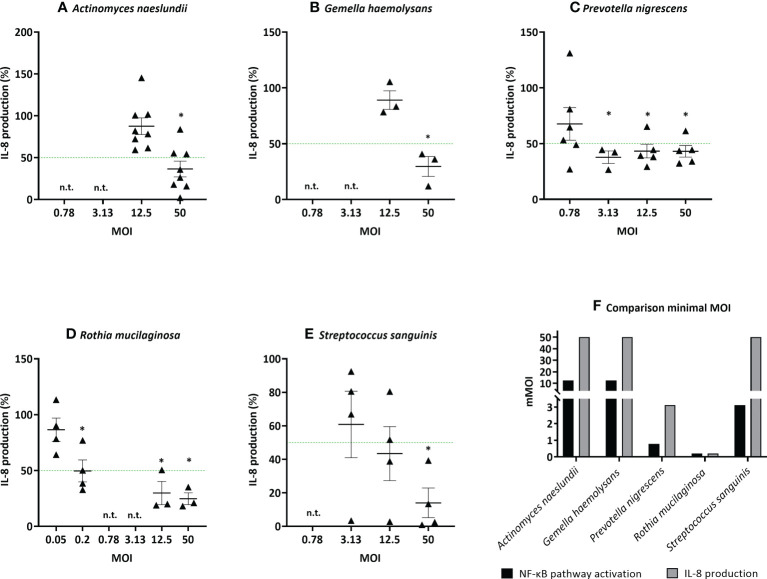
Determination of the minimal effective MOI (mMOI_IL-8_) of commensal bacteria in (3D) A549 cells based on IL-8 secretion. Selected lung commensals at different MOI were cultured with LPS and the mMOI_IL-8_ was determined using an ELISA for IL-8 quantification, with **(A)** *A. naeslundii*, **(B)** *G. haemolysans*, **(C)** *P. nigrescens*, **(D)** *R. mucilaginosa*, **(E)** *S. sanguinis*. On the vertical axis % IL-8 production compared to LPS-stimulated cells is shown, on the horizontal axis the tested MOI is depicted. The mMOI_IL-8_ was defined as the lowest MOI with anti-inflammatory activity (i.e.< 50% IL-8 production compared to LPS-stimulated cells as visualized by a green line, p< 0.05). *: p< 0.05, n ≥ 3, n.t.: not tested. **(F)** Comparison of the minimal MOI for inhibition of NF-κB pathway activation and IL-8 production. On the vertical axis the minimal MOI is shown, on the horizontal axis the selected microbiota members are depicted. Anti-inflammatory activity was defined as:< 50% NF-κB pathway activation/IL-8 secretion compared to LPS-stimulated cells, p< 0.05.

The 5 most potent microbiota members (based on the lowest mMOI_NF-κB_, **Figure 4**), each belonging to different genera, were further validated using an alternative measure of anti-inflammatory activity, i.e. based on the inhibition of IL-8 production. This inflammatory cytokine is upregulated by NF-κB-pathway activation and induces inflammation by attracting neutrophils to the inflammatory site in patients with chronic lung disease (38, 39). These species were *A. naeslundii* (mMOI_NF-κB_ = 12.5)*, G. haemolysans* (mMOI_NF-κB_ = 12.5)*, P. nigrescens* (mMOI_NF-κB_ = 0.78)*, R. mucilaginosa* (mMOI_NF-κB_ = 0.2), and *S. sanguinis* (mMOI_NF-κB_ = 3.13). Similarly to the NF-kB-based mMOI determination, the mMOI_IL-8_ was defined as the lowest dose that significantly reduced LPS-triggered inflammation (i.e. IL-8 production) by at least 50%. The mMOI_IL-8_ was 50 for *A. naeslundii*, 50 for *G. haemolysans*, 3.12 for *P. nigrescens*, 0.2 for *R. mucilaginosa* and 50 for *S. sanguinis* (**Figures 5A–E**). Hence, for all species, except *R. mucilaginosa*, a higher MOI was needed to reduce IL-8 production compared to inhibition of the NF-κB-pathway activation (**Figure 5F**).

### Anti-inflammatory species can reduce IL-8 production triggered by H_2_O_2_

Next, we evaluated whether the most potent anti-inflammatory bacteria (*A. naeslundii, G. haemolysans, P. nigrescens, R. mucilaginosa, S. sanguinis*) were able to inhibit inflammation in 3D A549 lung cells caused by another pro-inflammatory stimulus, i.e. H_2_O_2_, which is relevant for COPD (40–42). Hydrogen peroxide is a ROS, which is elevated in people living with COPD (pwCOPD) due to exogenous pollutants and inflammation leading to an oxidative imbalance (43, 44). In this study, hydrogen peroxide did not give a measurable signal when activation of the NF-κB pathway was used as read out (**Supplementary Figure S2**). Most likely, the NF-κB luciferase assay was not sensitive enough to detect H_2_O_2_-induced NF-κB pathway activation at the tested concentration or the chemical interfered with the read-out of the assay. Therefore, anti-inflammatory activity was evaluated by measuring IL-8 production after 3D lung cells were exposed to 1 mM H_2_O_2_ with or without single species at their mMOI_IL-8_.

**Figure 6:**
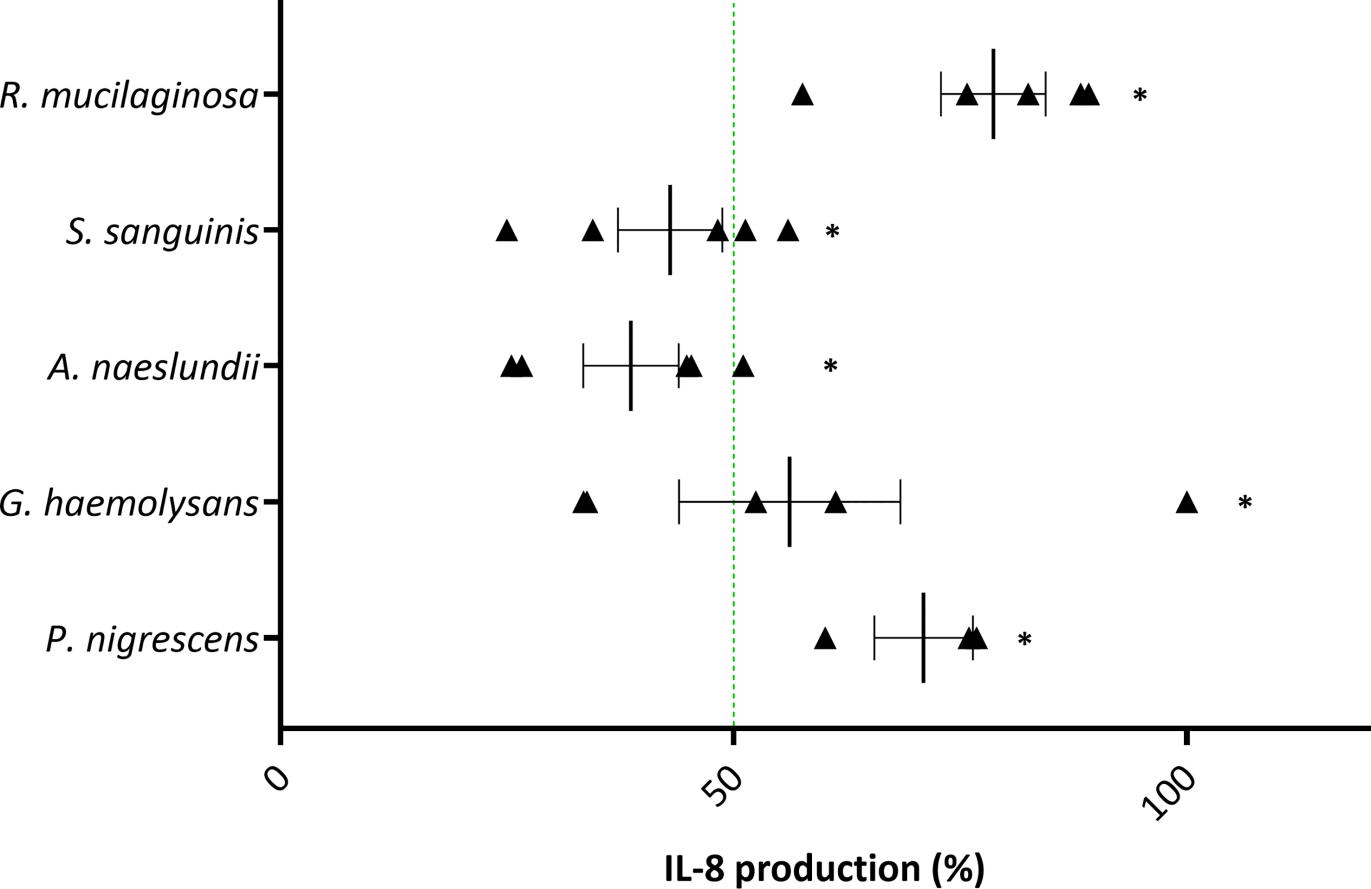
Anti-inflammatory activity of selected lung commensals with 1 mM H_2_O_2_ as pro-inflammatory stimulus in (3D) A549 cells based on IL-8 secretion. Selected lung commensals at their mMOI_IL-8_, in presence of H_2_O_2_, were evaluated for anti-inflammatory activity using an ELISA for IL-8 quantification. On the vertical axis the tested species are shown. On the horizontal axis the % IL-8 production compared to H_2_O_2_-stimulated cells is shown. Anti-inflammatory activity was defined as:< 50% IL-8 production compared to H_2_O_2_-stimulated cells as visualized by a green line, p< 0.05. *: p< 0.05, n ≥ 3.

All five anti-inflammatory bacteria significantly reduced IL-8 production triggered by H_2_O_2_ (**Figure 6**). Nevertheless, only *A. naeslundii* and *S. sanguinis* reduced inflammation by more than 50%. No significant cytotoxicity was observed for all five single species (**Supplementary Figure S3A**). However, the viability in presence of *A. naeslundii* or *G. haemolysans* was lower than 80% but not significantly different from the control. In addition, H_2_O_2_ had no influence on bacterial association with 3D lung epithelial cells (**Supplementary Figure S3B**).

### Synergistic activity is observed between anti-inflammatory species

**Figure 7:**
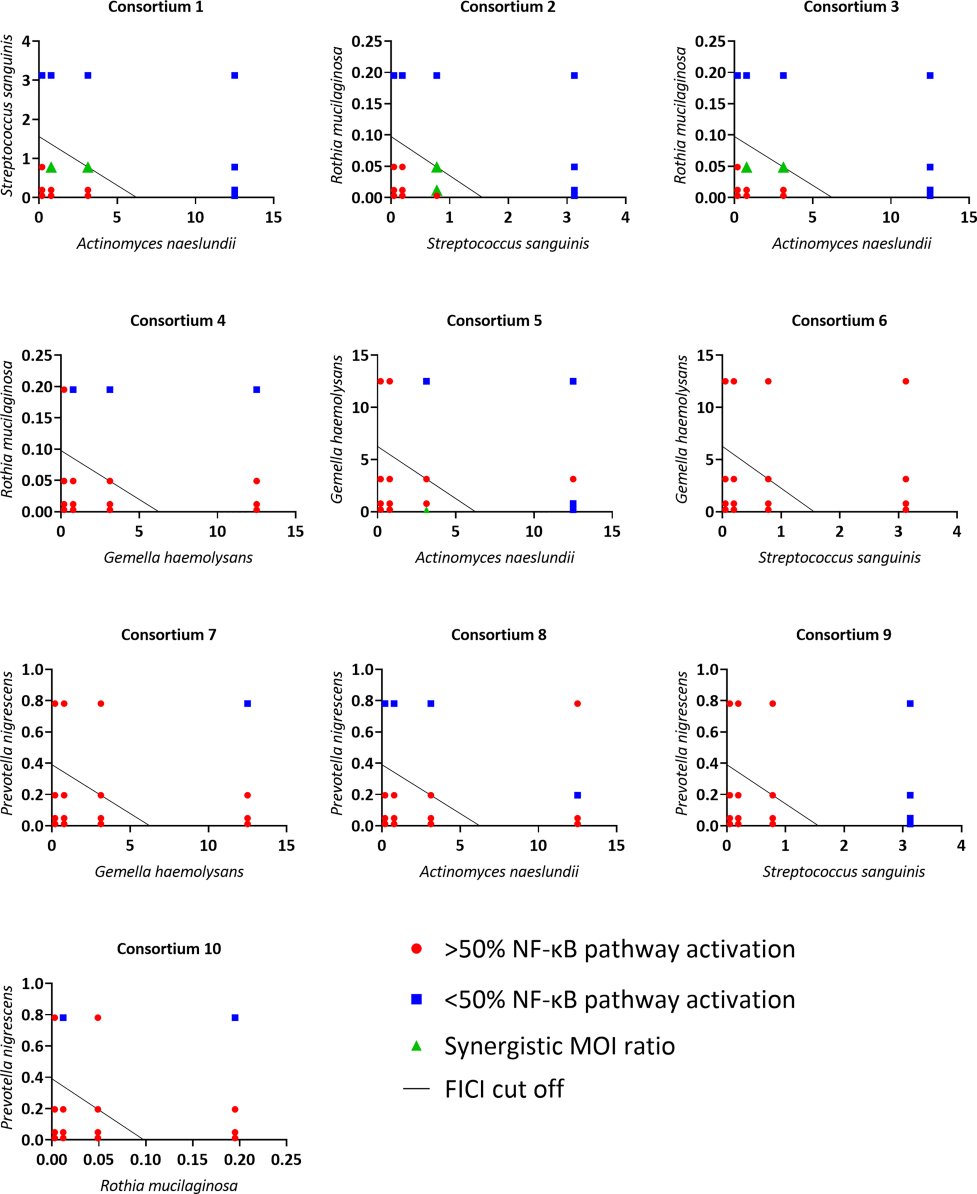
Isoboles of all tested consortia to visualize synergistic anti-inflammatory activity based on NF-κB pathway activation. On the vertical axis the tested MOI of one commensal species is shown, on the horizontal axis the tested MOI of the other commensal species is shown. MOI ratios were defined as synergistic if:< 50% NF-κB pathway activation compared to LPS-stimulated cells, p< 0.05 and FICI ≤ 0.5. n ≥ 3.

To evaluate synergistic activity between anti-inflammatory bacteria, the 5 most potent anti-inflammatory microbiota members (based on the lowest MOI_NF-κB_, **Figure 4**) belonging to different genera were used. We hypothesized that when two bacterial species are added simultaneously as a consortium, a lower total dose is needed to obtain an anti-inflammatory effect as compared to the dose needed using single species. To experimentally test this hypothesis, a checkerboard assay was done for each consortium comprised of two anti-inflammatory species. Each checkerboard contained 16 different MOI ratios, and FICI values were calculated for all MOI ratios. These FICI values were graphically visualized with an isobologram (**Figure 7**). The isobologram method is commonly used for assessing drug synergism and is similar to the FICI formula (45). In the present study, the axes on each isobologram represent the tested MOI of species A and species B. Hence, data points in the isobolograms represent the tested MOI ratios (i.e. 16 in total), while the line represents the FICI cut-off value for synergism (i.e. FICI ≤ 0.5) (46). When FICI ≤ 0.5, dots are located below or on the FICI cut-off, while values are displayed above the FICI cut-off for FICI > 0.5. For each consortium, the effect of each MOI ratio was evaluated based on LPS-triggered NF-κB pathway activation. When the MOI ratio was situated below or on the FICI cut-off and at least 50% inactivation of the NF-κB pathway was observed, a synergistic anti-inflammatory activity was exerted by that specific consortium at that particular MOI ratio, as compared to single bacterial species.

Synergistic MOI ratios within the consortium were labeled in green (**Figure 7**). Four out of ten tested consortia were synergistic as at least one MOI ratio had synergistic anti-inflammatory activity. For these synergistic consortia, FICI values and percentage of NF-κB pathway activation of the synergistic MOI ratios are shown in **Table 2**. These consortia were: *A. naeslundii* with *S. sanguinis* (consortium 1), *S. sanguinis* with *R. mucilaginosa* (consortium 2), *A. naeslundii* with *R. mucilaginosa* (consortium 3) and *A. naeslundii* with *G. haemolysans* (consortium 5).

**Table 2:** Synergistic consortia and single species with corresponding MOI ratios, NF-κB pathway activation compared to LPS-stimulated cells and FICI values.

**Consortium 1**				**Single species from consortium 1**			
***A. naeslundii*** **MOI_consortia_**	***S. sanguinis*** **MOI_consortia_**	**NF-κB pathway** **activation (%)**	**FICI**	***A. naeslundii*** **MOI_single_**	**NF-κB pathway** **activation (%)**	***S. sanguinis*** **MOI_single_**	**NF-κB pathway** **activation (%)**
**0.78**	**0.78**	**43.29**	**0.31**	**12.5**	**27.81**	**3.13**	**31.75**
**3.13**	**0.78**	**41.08**	**0.50**				
**Consortium 2**				**Single species from consortium 2**			
***S. sanguinis*** **MOI_consortia_**	***R. mucilaginosa*** **MOI_consortia_**	**NF-κB pathway** **activation (%)**	**FICI**	***S. sanguinis*** **MOI_single_**	**NF-κB pathway** **activation (%)**	***R. mucilaginosa*** **MOI_single_**	**NF-κB pathway** **activation (%)**
**0.78**	**0.01**	**47.51**	**0.31**	**3.13**	**31.75**	**0.20**	**44.96**
**0.78**	**0.05**	**47.02**	**0.50**				
**Consortium 3**				**Single species from consortium 3**			
***A. naeslundii*** **MOI_consortia_**	***R. mucilaginosa*** **MOI_consortia_**	**NF-κB pathway** **activation (%)**	**FICI**	***A. naeslundii*** **MOI_single_**	**NF-κB pathway** **activation (%)**	***R. mucilaginosa*** **MOI_single_**	**NF-κB pathway** **activation (%)**
**0.78**	**0.05**	**41.53**	**0.31**	**12.5**	**27.81**	**0.20**	**44.96**
**3.13**	**0.05**	**32.00**	**0.50**				
**Consortium 5**				**Single species from consortium 5**			
***A. naeslundii*** **MOI_consortia_**	***G. haemolysans*** **MOI_consortia_**	**NF-κB pathway** **activation (%)**	**FICI**	***A. naeslundii*** **MOI_single_**	**NF-κB pathway** **activation (%)**	***G. haemolysans*** **MOI_single_**	**NF-κB pathway** **activation (%)**
**3.13**	**0.20**	**42.88**	**0.27**	**12.5**	**27.81**	**12.5**	**30.56**

MOI ratios were defined as synergistic if: < 50% NF-κB pathway activation compared to LPS-stimulated cells, p < 0.05 and FICI ≤ 0.5. n ≥ 3. Single species from the corresponding consortia with their mMOI_NF-κB_ and NF-κB pathway activation compared to LPS-stimulated cells are presented in the right column. The mMOI_NF-κB_ was defined as the lowest MOI with anti-inflammatory activity (i.e. < 50% NF-κB pathway activation compared to LPS-stimulated cells, p < 0,05, n ≥ 3).

Since we made dilutions starting from the mMOI_NF-κB_ of each microbiota member, a maximum of 9/16 MOI ratios could in theory be synergistic according to the FICI formula. However, only 2/9 MOI ratios were defined as synergistic for consortium 1, 2 and 3, and only one MOI ratio for consortium 5. The cut-off (i.e. 50% reduction in NF-κB pathway activation) that we defined as criterium for anti-inflammatory activity could be an explanation for this observation. Indeed, many other synergistic MOI ratios (i.e. FICI ≤ 0,5) from consortium 1, 2, 3 and 5 also had a significant reduction in NF-κB pathway activation but this was less than 50% (**Supplementary Table 1**).

**Figure 8:**
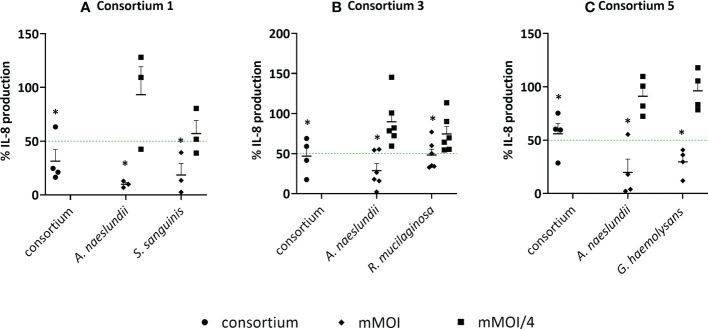
Anti-inflammatory activity of synergistic consortia and single species in (3D) A549 cells based on IL-8 secretion. Single species at their mMOI_IL-8_ and mMOI_IL-8_/4 and synergistic consortia at defined MOI ratios, in the presence of LPS, were evaluated for anti-inflammatory activity by using an ELISA for IL-8 quantification with **(A)** consortium 1; *A. naeslundii* with *S. sanguinis*, **(B)** consortium 3; *A. naeslundii* with *R. mucilaginosa*, **(C)** consortium 5; *A. naeslundii* with *G. haemolysans.* On the vertical axis, the % IL-8 production compared to LPS-stimulated cells is shown, on the horizontal axis the consortium and single species at selected MOI (i.e. mMOI_IL-8_ and mMOI_IL-8_/4) are presented. Anti-inflammatory (synergistic) activity was defined as:< 50% IL-8 production compared to LPS-stimulated cells as visualized by a green line, p< 0.05, and (FICI ≤ 0.5). *: p<0.05, n ≥ 3.

To validate if synergistic consortia, which were selected based on NF-κB pathway inactivation as a read out, could also reduce LPS-triggered IL-8 production in 3D A549 lung epithelial cells, three consortia were selected, i.e. *A. naeslundii* with *R. mucilaginosa*, *A. naeslundii* with *G. haemolysans* and *A. naeslundii* with *S. sanguinis*. As mentioned above, we observed that the mMOI differs when NF-κB pathway activation (mMOI_NF-κB_) or IL-8 production (mMOI_IL-8_) are measured as an outcome (**Figure 5F**). Hence, we could not use synergistic MOI ratios as determined by the inhibition of the NF-κB pathway (**Table 2**) for validation purposes. Therefore, based on the definition by Odds et al. (2003) (36), we tested whether the mMOI_IL-8_/4 of both species is anti-inflammatory in a consortium but not in single species. In this case, a FICI ≤ 0,5 and hence synergism is achieved. As expected, all single species showed a significant reduction of LPS-triggered IL-8 production at their mMOI_IL-8_, which was not observed at an mMOI_IL-8_/4 (**Figure 8**). When the mMOI_IL-8_/4 of two species was combined in a consortium, more than 50% reduction in IL-8 production was observed compared to LPS-stimulated cells, for 2/3 consortia (i.e. consortium 1 and 3), while the consortium consisting of *A. naeslundii* and *G. haemolysans* (consortium 5) significantly reduced IL-8 production by 44% (p< 0.05). In addition, cytotoxicity of these three consortia was tested, and no significant LDH release was observed (**Supplementary Figure S4**).

## Discussion

In this study, we demonstrate that several non-pathogenic members of the lung microbiota have anti-inflammatory properties, based on their ability to inhibit NF-κB pathway activation and IL-8 secretion, induced by the pro-inflammatory triggers LPS or H_2_O_2_ in a physiologically relevant 3D lung epithelial cell model. In addition, synergistic anti-inflammatory activity was observed between species of the lung microbiota.

The bacterial species that were evaluated for anti-inflammatory activity in this study were isolated from sputum samples of pwCF. While bronchoalveolar lavage (BAL) is the golden standard to sample the lower airways, less invasive methods (such as spontaneously expectorated sputum) are often preferred (47). Nevertheless, the used sampling method may imply the inclusion of some commensals of the oral cavity, which was minimized by rinsing sputum samples with physiological saline prior to bacterial isolation to remove saliva. Interestingly, the species that are part of the CF lung microbiota are highly comparable with those found in other chronic lung diseases, with some differences such as a higher *Streptococcus* abundance in pwCOPD (48). In addition, interpatient variability in the relative abundance of many genera of the lung microbiota has been reported for patients with different chronic lung diseases (8, 20, 49–51). In this study, we isolated 29 species from 9 non-pathogenic genera (i.e. *Actinomyces, Atopobium, Gemella, Micrococcus, Neisseria, Prevotella, Rothia, Streptococcus* and *Veillonella*). Similarly, another study on sputum samples of pwCF, isolated 21 commensal species from 7 genera, and the genus *Streptococcus* was widely represented with 10 different species present (20). In the present study, 7 different *Streptococcus* species were identified. The identified genera and selected species represent a significant part of the lung microbiota in chronic lung diseases.

We observed that none of the tested species induced inflammation by itself, while 9/15 non-cytotoxic lung commensals could inhibit NF-κB pathway activation after stimulation with pro-inflammatory stimuli relevant for chronic lung diseases (LPS or H_2_O_2_). Recent studies have described beneficial properties of some microbiota members that were identified as anti-inflammatory in the present study. Specifically, anti-inflammatory activity was previously described for *Prevotella*, *Rothia* and *Streptococcus* species (19–21, 52). Recently, *P. nigrescens* and *P. histicola* were reported to diminish NF-κB (p65) signaling and levels of cytokines, such as IL-6 and IL-8, induced by *P. aeruginosa* in CF airway epithelial cells (CFBE41o- cells) by reducing *P. aeruginosa* growth (53). For the genus *Streptococcus*, various species, such as *S. mitis*, *S. oralis* and *S. cristatus*, reduced inflammation triggered by *P. aeruginosa* in BEAS-2B cells (20). Another study found that the majority of oral streptococci was able to reduce CXCL8 secretion through inhibition of NF-κB pathway activation in 16HBE14o- epithelial cells, stimulated with flagellin (54). As mentioned above, *Rothia* species, incubated under aerobic or microaerobic conditions (3% O_2_, 5% CO_2_ and 92% N_2_, 37°C), were found to interfere with NF-κB pathway activation *in vitro*, and showed anti-inflammatory activity *in vivo* as well (19). In the present study, *R*. *mucilaginosa* was found to be the most potent anti-inflammatory species since a very low dose (i.e. MOI 0.2) reduced NF-κB pathway activation and IL-8 production when 3D lung cells were triggered with both LPS or H_2_O_2_. Moreover, many studies have linked the relative abundance of *Rothia* spp. in the airways with lower levels of inflammatory markers (i.e. IL-8, IL-6 and IL-1β) in chronic lung diseases (55, 56). Even the relative abundance of *Rothia* in the gut was shown to have an influence on asthma predisposition (57, 58). For *Gemella* spp., a study investigating the microbiome profiles of the different inflammatory phenotypes in severe asthma patients, found that the abundance of *Gemella* spp. was negatively associated with the percentage of sputum neutrophils in patients with neutrophilic asthma (59). While for *Actinomyces* spp. no studies were found on anti-inflammatory properties, other beneficial characteristics were reported, including inhibition of biofilm formation, virulence and proliferation of *Candida albicans* (60).

Interestingly, the minimal dose where anti-inflammatory activity was observed differed between genera but also between species of the same genus. Possible reasons for this divergence might be a difference in the type and amount of effector molecules produced by the different bacterial species. Further research, including metabolomic analysis, is now needed to narrow this knowledge gap as anti-inflammatory mediators of the lung microbiota have not been reported yet. In addition, when comparing the mMOI necessary to reduce IL-8 secretion or NF-kB pathway activation in 3D lung epithelial cells, a higher mMOI_IL-8_ was typically needed, with the exception of *R. mucilaginosa* where an equal mMOI_IL-8_ and mMOI_NF-κB_ was obtained. A possible explanation for this finding is that a higher net bacterial load may be needed to reduce IL-8 secretion since cytokine production is located downstream in the LPS-triggered inflammatory process compared to activation of the NF-κB pathway (38, 61). Additionally, IL-8 production triggered by LPS stimulation may result from activation of other inflammatory pathways besides the NF-κB pathway, such as mitogen-activated protein kinase (MAPK) (62–64), extracellular signal-regulated kinase (ERK) (65) and c-Jun N-terminal kinase (JNK) (66). The involvement of different inflammatory pathways could also explain the lower reduction of IL-8 secretion when H_2_O_2_ was used as pro-inflammatory stimulus compared to LPS. For example, ROS levels can activate the ROS–NLRP3–caspase-1 pathway in A549 cells, in addition to the NF-κB pathway (67).

Another finding in this study is that differences in cytotoxicity were observed between different genera of the lung microbiota but also within a single genus. Specifically, *S. sanguinis* did not exhibit cytotoxicity, while *S. salivarius* was strongly cytotoxic. Controversially, *S. salivarius* is often described in literature as an anti-inflammatory commensal, and has been studied as a probiotic against otitis media in several studies (68, 69). For example, *S. salivarius* K12 was tested *in vitro* for its anti-inflammatory effects in 16HBE14o- cells and showed no cytotoxicity using the LDH assay (70). To our knowledge, the present study is the first describing cytotoxic effects of *S. salivarius*. However, cytotoxicity was reported for other oral streptococci, such as *S. mitis* and *S. cristatus*, which caused moderate LDH release when cultured with 16HBE14o- cells for 24h (54). Differences between previous studies and the present study can potentially be explained by the use of different *S. salivarius* strains since most probiotic studies investigated *S. salivarius* strain K12 (69). In addition, other host cell types, growth conditions and the type of LDH assay could have an influence (32, 54). In this study, microaerobic conditions (3% O_2_, 5% CO_2_ and 92% N_2_) were used during incubation, which resembles the diseased lung environment more closely since low oxygen levels have been reported in the viscous mucus (71). However, most studies use aerobic conditions (54, 70) for *in vitro* co-culture experiments. Moreover, previous research demonstrated that *S. maltophilia* and *S. pneumoniae* interfered with the outcome of the LDH assay by either protease activity or acidification of cell supernatant (32). For that reason, an alternative LDH assay was used in the present study, which avoids potential underestimation of LDH activity by bacterial interference (32).

Next, we demonstrated that lung commensals can synergistically interact with each other to influence host inflammation. The data in this study reveal that synergism can occur but that it is not common. Here, 4/10 studied consortia were found to have synergistic anti-inflammatory activity for inhibition of NF-κB pathway activation. In addition, inhibition of IL-8 production in LPS-stimulated 3D A549 lung epithelial cells was also demonstrated for the three evaluated synergistic consortia. It was noted that three out of four synergistic consortia contained *Actinomyces*. We hypothesize that the target of *Actinomyces* in the NF-κB pathway might be different than that of the other four commensals, which may result in a more pronounced inactivation of NF-κB signaling when combining *Actinomyces* with any one of the other tested species.

In conclusion, our data demonstrate that the lung microbiota contains various anti-inflammatory species and that the minimal dose to exert an effect *in vitro* may differ between species. Furthermore, synergism between anti-inflammatory species was found, which brings forward a possible role of interspecies interactions in the net inflammatory response of the host.

## Data availability statement

The original contributions presented in the study are publicly available. This data can be found here: Genbank [SUB12991115 P1_AE4 OQ693609; SUB12991115 P4_MA3 OQ693610].

## Ethics statement

The local ethics committee of the Ghent University Hospital approved the study (registration number B670201836204). Written informed consent to participate in this study was provided by the participants’ legal guardian/next of kin.

## Author contributions

AC, EG and CR conceptualized the study and designed the experimental set-up. EB and YV provided the sputum samples of pwCF. LG and SB isolated and identified the lung microbiota members. EG performed the experiments and data analysis. TM and KB provided guidance in the experimental set-up. EG and AC wrote the manuscript, with input from all authors. All authors contributed to the article and approved the submitted version.
