# Supplementary material for: Commensal bacteria of the lung microbiota synergistically inhibit inflammation in a three-dimensional epithelial cell model
Source: Front Immunol. 2023 Apr 21;14:1176044. doi: 10.3389/fimmu.2023.1176044 (PMC10164748; doi:10.3389/fimmu.2023.1176044)
Supplement: Supplementary file 1 [file DataSheet_1.docx]

Supplementary Material

Commensal bacteria of the lung microbiota synergistically inhibit inflammation in a three-dimensional epithelial cell model

***Ellen Goeteyn^1^, Lucia Grassi^1^, Sara Van den Bossche^1^, Charlotte Rigauts^1^, Yannick Vande Weygaerde^2^, Eva Van Braeckel^2,3^, Tania Maes^3^, Ken R Bracke^3^, and Aurélie Crabbé^1^.***


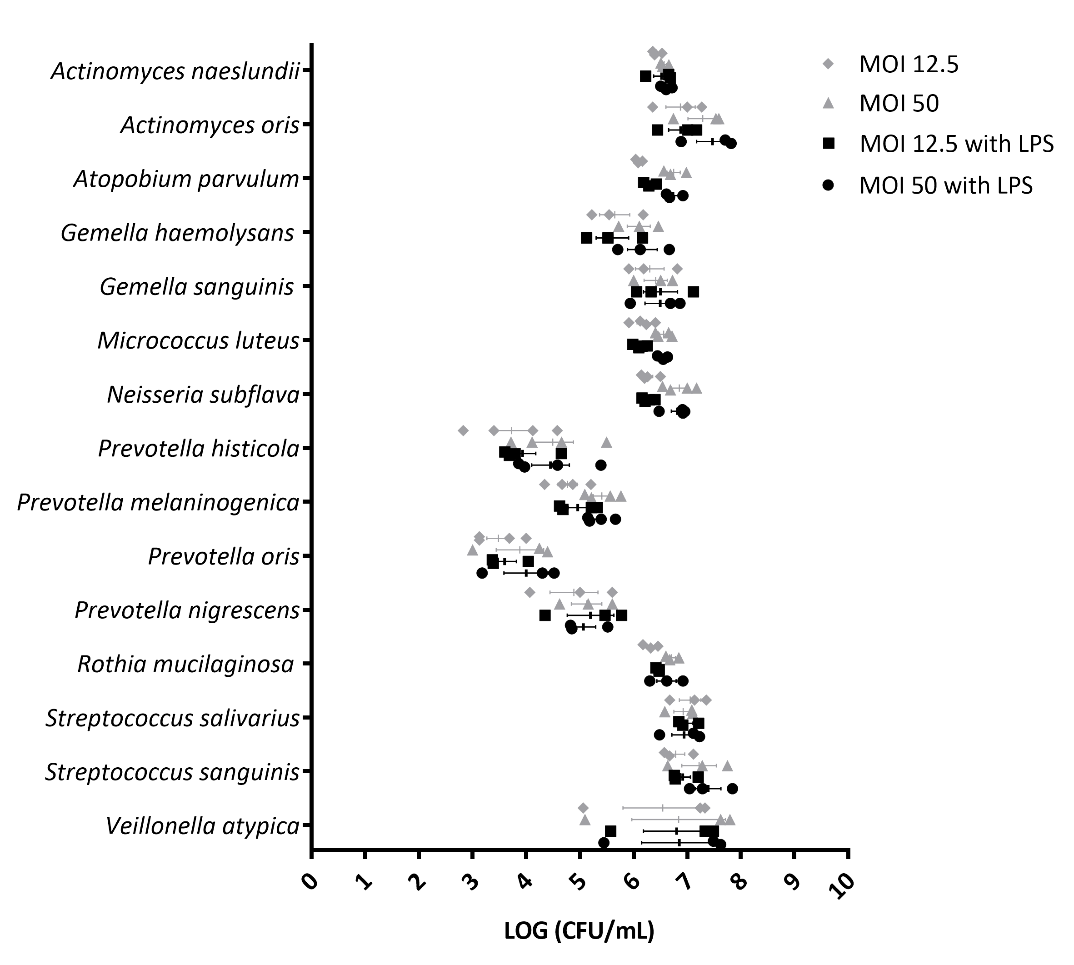


**Supplementary Figure 1.** Association of commensal species with 3D A549 lung epithelial cells in the presence and absence of LPS. Isolated lung commensals were tested at an MOI of 50 and an MOI of 12.5, with or without LPS. On the vertical axis, lung commensals are depicted, on the horizontal axis, LOG (CFU/mL) values are shown. No significant difference was observed when commensals were co-cultured with or without LPS. n ≥ 3.


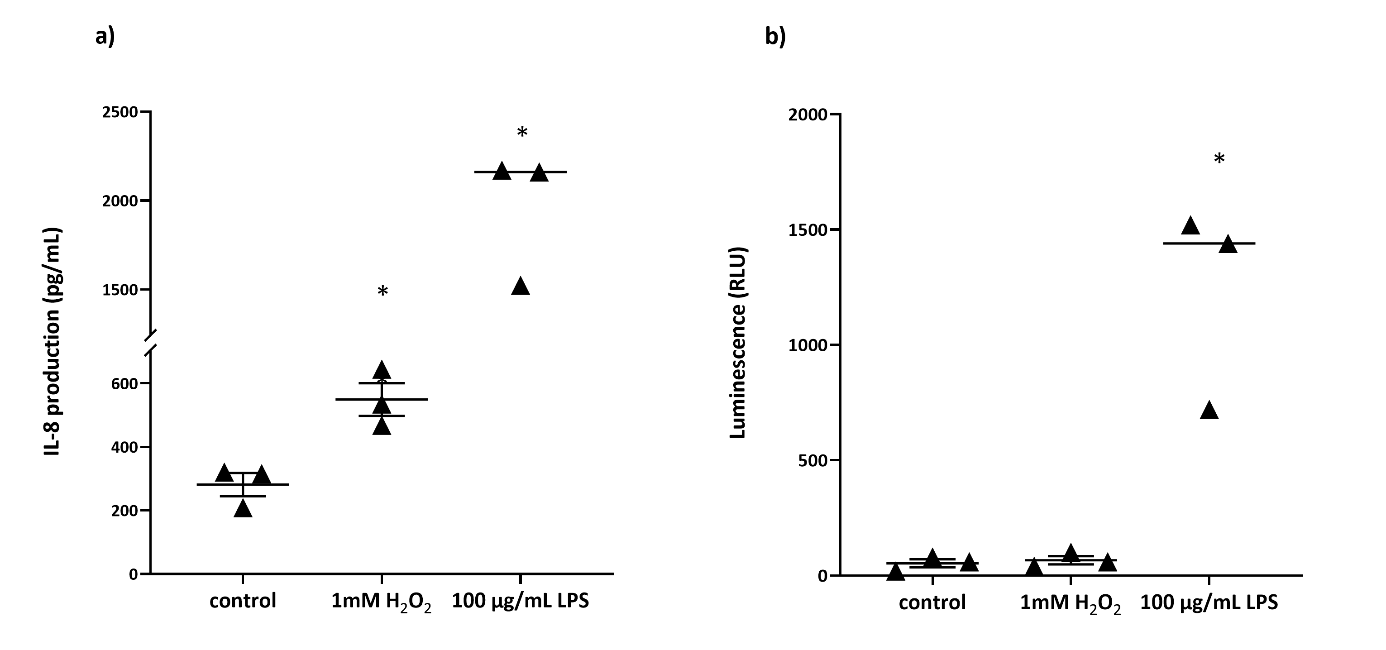


**Supplementary Figure 2.** Evaluation of inflammation in (3D) A549 (luciferase NF-κB-reporter) cells triggered with 1 mM H_2_O_2_ or 100 µg/mL LPS. (a) IL-8 production (pg/mL) when cells were incubated with or without pro-inflammatory stimuli (i.e. 1 mM H_2_O_2_, 100 µg/mL LPS) . *: p < 0.05, N = 3. (b) Activation of the NF-κB pathway was measured via luminescence (RLU) when cells were incubated with or without pro-inflammatory stimuli (i.e. 1 mM H_2_O_2_, 100 µg/mL LPS). *: p < 0.05, N = 3.


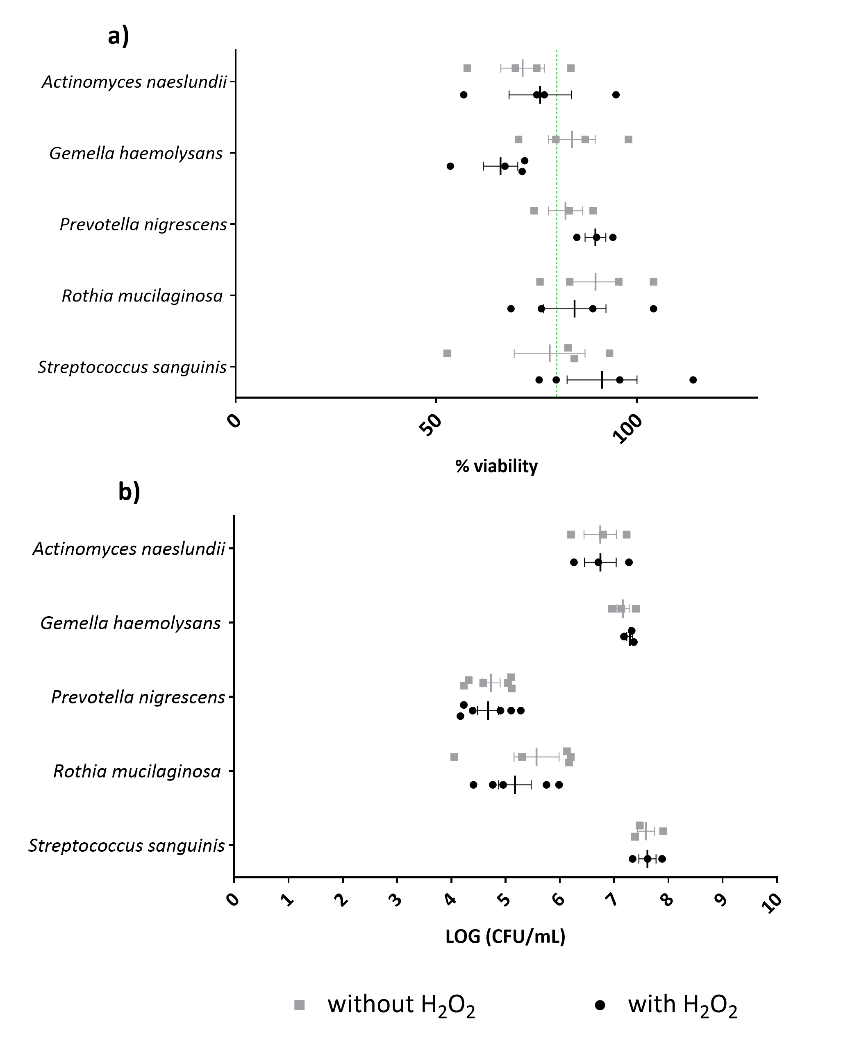


**Supplementary Figure 3**. Cytotoxicity and bacterial association to lung epithelial cells of lung microbiota members in (3D) A549 cells with 1 mM H_2_O_2_ as pro-inflammatory stimulus. (a) Selected lung microbiota members at their mMOI_IL-8_, with or without H_2_O_2,_ were evaluated for cytotoxicity by a LDH assay. On the vertical axis selected lung commensals are depicted. On the horizontal axis, the % viability compared to cells cultured alone is shown. Cytotoxic effects were defined as: < 80% viability compared to cells cultured alone as visualized by a green line, p < 0.05. No significant difference was observed. n ≥ 3. (b) Selected lung microbiota members at their mMOI_IL-8_, with or without H_2_O_2,_ were evaluated for bacterial association to lung epithelial cells. On the vertical axis, selected lung commensals are depicted. On the horizontal axis, LOG (CFU/mL) values are shown. No significant difference was observed when commensals were co-cultured with or without H_2_O_2,_. n ≥ 3.


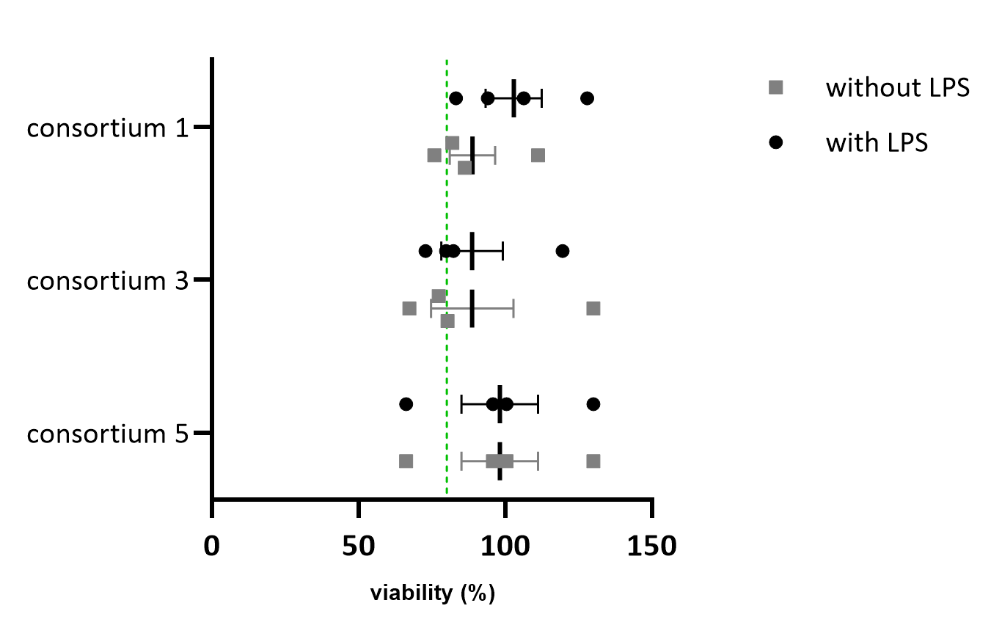


**Supplementary Figure 4**. Cytotoxicity of synergistic consortia in (3D) A549 cells. Synergistic consortia at defined MOI ratios, with or without LPS, were evaluated for cytotoxicity by using a LDH assay with consortium 1 (*A. naeslundii* with *S. sanguinis*), consortium 3 (*A. naeslundii* with *R. mucilaginosa*) and consortium 5 (*A. naeslundii* with *G. haemolysans*). On the vertical axis the three consortia are depicted. On the horizontal axis, the % viability compared to cells cultured alone is shown. Cytotoxic effects were defined as: < 80% viability compared to cells cultured alone as visualized by a green line, p < 0.05. No significant difference was observed. n ≥ 3.

**Supplementary Table 1**. Synergistic consortia with all tested MOI ratios, NF-kB pathway activation compared to LPS-stimulated cells, FICI and p values. MOI ratios were defined as synergistic if: < 50% NF-κB pathway activation compared to LPS-stimulated cells, p < 0.05 and FICI ≤ 0.5. n ≥ 3. Synergistic MOI ratios are indicated in bold.
